# Mapping the Comorbidome in Chronic Obstructive Pulmonary Disease: Prevalence and Mortality Risk in a Colombian Cohort

**DOI:** 10.3390/jcm15093365

**Published:** 2026-04-28

**Authors:** Charbel Kamil Faizal Gómez, Eduardo Tuta Quintero, Alirio Rodrigo Bastidas, Alejandra Lozano Forero, Miguel David Nieto González, Valentina Ortíz Marquez, María José Herran Pérez, Ana Carolina Colmenares Leal, Mateo Mariño Rodríguez, Juan Camilo Rodríguez Sánchez, David Sebastián Cárdenas Rodríguez, Fulton Camilo Perea Gómez, Andrés Felipe Cardona Arango, Tomás Salamanca España, Juan David Pardo Gallego, José Raul Restrepo Garzón

**Affiliations:** 1 School of Medicine, Universidad de La Sabana, Campus del Puente del Común, Km. 7 Autopista Norte de Bogotá, Chía 250001, Cundinamarca, Colombia; charbelfago@unisabana.edu.co (C.K.F.G.); alejandralofo@unisabana.edu.co (A.L.F.); miguelnigo@unisabana.edu.co (M.D.N.G.); valentinaorma@unisabana.edu.co (V.O.M.); mariaherpe@unisabana.edu.co (M.J.H.P.); anacole@unisabana.edu.co (A.C.C.L.); mateomaro@unisabana.edu.co (M.M.R.); juanrodrsan@unisabana.edu.co (J.C.R.S.); andrescarar@unisabana.edu.co (A.F.C.A.); tomassaes@unisabana.edu.co (T.S.E.); juanpargal@unisabana.edu.co (J.D.P.G.); joseresga@unisabana.edu.co (J.R.R.G.); 2 Master’s Candidate in Epidemiology, School of Medicine, Universidad de La Sabana, Campus del Puente del Común, Km. 7 Autopista Norte de Bogotá, Chía 250001, Cundinamarca, Colombia; 3 Resident of Internal Medicine, School of Medicine, Universidad de La Sabana, Campus del Puente del Común, Km. 7 Autopista Norte de Bogotá, Chía 250001, Cundinamarca, Colombia; eduardotuqu@unisabana.edu.co (E.T.Q.); davidcarro@unisabana.edu.co (D.S.C.R.); fultonpego@unisabana.edu.co (F.C.P.G.)

**Keywords:** Chronic Obstructive Pulmonary Disease, comorbidities, comorbidome, mortality, cohort

## Abstract

**Background/Objectives**: Chronic Obstructive Pulmonary Disease (COPD) is frequently associated with multiple comorbidities that influence prognosis. The comorbidome is a graphical representation of both the prevalence and strength of association of each comorbidity with COPD, allowing rapid identification of the most relevant risk factors. The aim of this study was to evaluate the association between comorbidities and mortality in patients with COPD using a comorbidome approach. **Methods**: We conducted a retrospective cohort study of 500 patients aged ≥40 years with COPD treated between 2005 and 2020 at Clínica Universidad de La Sabana (Chía, Colombia). Demographic variables, comorbidities, and mortality were recorded. The prevalence of each comorbidity was expressed as a percentage, and their association with mortality was assessed using odds ratios (OR) derived from univariate contingency tables with 95% confidence intervals (95% CI). The comorbidome was constructed by plotting the inverse odds ratio (1/OR) against the prevalence of each condition. **Results**: The mean age was 76.6 years (SD 11.3). Overall mortality was 28.4%. The most prevalent comorbidities were hypertension (45.2%) and smoking (38%). Comorbidities significantly associated with mortality in unadjusted analyses included congestive heart failure (OR: 4.28; 95% CI: 2.55–7.18), arrhythmias (OR: 2.86; 95% CI: 1.60–5.13), acute myocardial infarction (OR: 2.58; 95% CI: 1.52–4.38), moderate or severe renal disease (OR: 2.08; 95% CI: 1.07–4.04), peripheral vascular disease (OR: 1.94; 95% CI: 1.10–3.40), and hypertension (OR: 1.66; 95% CI: 1.12–2.46). **Conclusions**: The most prevalent comorbidities were hypertension and smoking. However, the conditions significantly associated with mortality in unadjusted analyses were congestive heart failure, arrhythmias, acute myocardial infarction, moderate or severe renal disease, peripheral vascular disease, and hypertension.

## 1. Introduction

Chronic Obstructive Pulmonary Disease (COPD) is a heterogeneous and inflammatory respiratory condition characterized by persistent respiratory symptoms such as dyspnea, chronic cough, and sputum production, resulting from structural and functional changes in the airways and/or alveoli, which generate chronic and progressive airflow obstruction [1]. In male populations, the global prevalence is estimated at 15.7% (95% CI: 13.8–18.6%) [2] and it is the fourth leading cause of mortality worldwide, responsible for more than 3 million deaths annually [3].

The coexistence of COPD with cardiovascular diseases (CVD) is frequent and multifactorial. Both entities share risk factors such as smoking, physical inactivity, and unhealthy diet, which promote endothelial dysfunction and systemic inflammation, contributing to the development of atherosclerosis and heart failure [4,5]. In patients with COPD, the prevalence of hypertension reaches 56–64%, and congestive heart failure (CHF) occurs in up to 15% of cases, increasing mortality and hospitalizations [5,6]. Chronic hypoxemia favors myocardial fibrosis and the onset of arrhythmias, while ventricular dysfunction reduces muscle perfusion, which worsens exercise intolerance and intensifies dyspnea [7,8]. In addition, autonomic neuropathy secondary to sustained hypoxemia can alter heart rate variability and prolong the QT interval, thereby increasing the risk of arrhythmias [9,10].

Beyond CVD, other comorbidities have been described. On the one hand, osteoporosis affects up to 38% of patients with moderate to severe disease, increasing the risk of fractures and impairing mobility [11]. On the other hand, diabetes mellitus, with a prevalence of 16%, worsens metabolic control and is associated with a higher number of exacerbations [12]. Similarly, mood disorders such as anxiety and depression may affect approximately 25–40% of patients, reducing treatment adherence and worsening quality of life [13].

In Colombia, evidence regarding the relationship between COPD and the prevalence of comorbidities is limited. The comorbidity map (Comorbidome) is a valuable tool to understand the comorbidities associated with COPD, being essential to address patients and stratify risk. Therefore, the main objective of this study is to determine the prevalence of comorbidities in patients diagnosed with COPD in Colombia and describe those most frequently associated using the Comorbidome.

## 2. Materials and Methods

A retrospective cohort study was conducted in subjects treated at a tertiary care center from 2005 to 2020. The main objective of the study was to describe the comorbidities most frequently associated with COPD and represent them in a comorbidity map (Comorbidome) reflecting their prevalence. The presentation of this study adheres to the STROBE guidelines [14].

### 2.1. Eligibility Criteria

Patients over 40 years of age with a diagnosis of COPD, defined by a post-bronchodilator FEV1/FVC <0.7 measured by spirometry according to GOLD criteria [15], were included. Patients without follow-up survival information, without spirometry data, or with a history of pulmonary diseases other than COPD were excluded.

### 2.2. Variables and Data Collection

Sociodemographic variables, past medical history, and smoking history were analyzed. Data collection was carried out using a database obtained directly from electronic medical records and diagnostic test results. To determine patients’ follow-up status, the Administrator of Health System Resources (ADRES) was consulted to report survival status. Precise eligibility criteria were established to mitigate selection bias. To reduce transcription bias, at least two team members reviewed the information, and in cases of inconsistency, a third team member reviewed the information and made the final decision. In addition, all variables were clearly defined, and the researchers responsible for data collection were previously trained.

All patients who met the inclusion criteria during the study period were included. Moderate or severe kidney disease was defined according to Charlson Comorbidity Index criteria, based on clinical record data. Patients were followed from the time of COPD diagnosis until death or the end of the study period, based on available clinical records and mortality data. Smoking was included as a clinical exposure variable given its relevance in COPD populations.

### 2.3. Sample Size

All patients who met the inclusion criteria during the study period were included.

### 2.4. Statistical Analysis

Data were transcribed into the Research Electronic Data Capture (REDCap) software version 13.1.4 [16]. Quantitative variables were summarized using measures of central tendency and dispersion, with means and standard deviations (SD) for normally distributed variables, and medians and interquartile ranges for non-normal distributions. The Shapiro–Wilk test was used to assess normality. Qualitative variables were described as absolute and relative frequencies. A univariate analysis was performed between the study variables and the presence or absence of mortality in patients with COPD using the chi-square test for qualitative variables and Student’s t-test or Mann–Whitney U test depending on the distribution of quantitative variables. For comorbidities with zero or very low frequency of events in one of the groups, odds ratios could not be estimated.

Subsequently, the prevalence of each comorbidity was calculated as a percentage together with the odds ratio and its 95% confidence interval. For the visualization of comorbidities associated with COPD, a comorbidome was created. This graphic represents all comorbidities with associations to COPD, if each comorbidity reflects a pathophysiological and epidemiological relationship. The size of each bubble is proportional to the prevalence of the disease in the cohort, and proximity to the center reflects the strength of the association, which was quantified numerically using the inverse odds ratio (1/OR). No imputation of missing data was performed; statistical analysis was conducted in Microsoft Excel [17], while data preparation and graphical visualizations were carried out with Python 3.12 [18].

### 2.5. Ethical Considerations

The study was conducted in accordance with the principles of the Declaration of Helsinki of 1975, as revised in 2024, as well as local, regional, and international regulations related to clinical research, including Colombian legislation on biomedical research. Ethical approval was granted by the Medical Ethics Committee of the center where the research was conducted (approval number: 20241104). All personal patient data have been deleted. This was a retrospective study; therefore, informed consent was not required.

## 3. Results

### 3.1. General Characteristics of the Population

**Figure 1:**
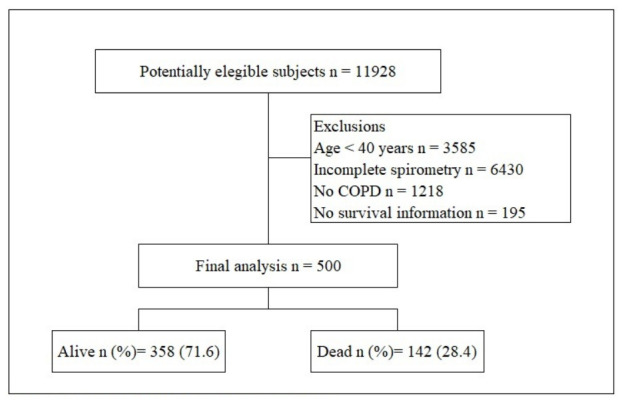
Enrollment of subjects in the study. COPD: Chronic obstructive pulmonary disease.

A total of 500 patients with COPD were included (Figure 1). The mean age was 76.6 years (SD 11.3); 85.6% (428/500) were older than 65 years, and 57.6% (288/500) were male (Table 1). Overall mortality was 28.4% (142/500), being significantly higher in older patients (82.6 vs. 74.3 years, *p* < 0.001) and in those with a history of hypertension (54.2% vs. 41.6%, *p* = 0.011), smoking (47.9% vs. 34.1%, *p* = 0.022), congestive heart failure (28.9% vs. 8.7%, *p* < 0.001), acute myocardial infarction (21.8% vs. 9.8%, *p* < 0.001), arrhythmias (18.3% vs. 7.3%, *p* < 0.001), peripheral vascular disease (16.9% vs. 9.5%, *p* = 0.020), and moderate or severe renal disease (12% vs. 6.1%, *p* = 0.028).

### 3.2. Prevalence of Comorbidities in COPD

In patients with COPD, the most prevalent comorbidities were hypertension, present in 45.2% (226/500), and smoking, with a prevalence of 38% (190/500). Other frequent comorbidities included hypothyroidism, with a prevalence of 18.4% (92/500), and congestive heart failure, present in 14.4% (72/500). Uncomplicated diabetes was observed in 13.4% (67/500), acute myocardial infarction in 13.2% (66/500), and asthma in 11.8% (59/500). Cases of peripheral vascular disease, arrhythmias, and moderate or severe renal disease were also recorded, with prevalences of 11.6% (58/500), 10.4% (52/500), and 7.8% (39/500), respectively (Table 2).

### 3.3. Association Between Comorbidities and Mortality

**Figure 2:**
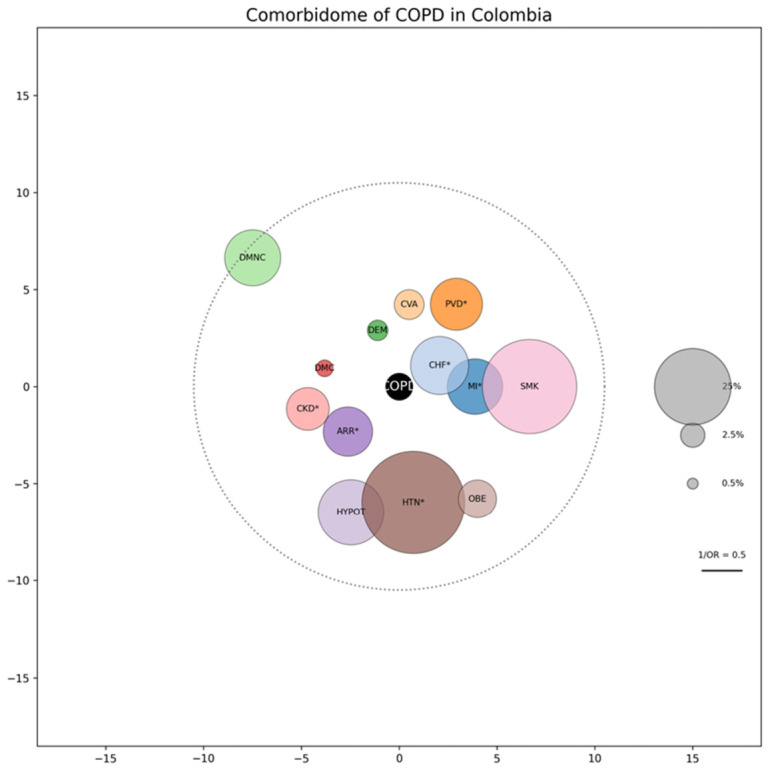
Comorbidome of COPD in Colombia. MI = Myocardial Infarction; CHF = Congestive Heart Failure; PVD = Peripheral Vascular Disease; CVA = Cerebrovascular Accident; DEM = Dementia; DMNC = Diabetes Mellitus without Complications; DMC = Diabetes Mellitus with Organ Damage; CKD = Chronic Kidney Disease (moderate or severe); ARR = Arrhythmias; HYPOT = Hypothyroidism; HTN = Hypertension; OBE = Obesity; PSY = Psychosis; SMK = Smoking. *: conditions that were statistically significant.

In patients with COPD, the comorbidities significantly associated with mortality in unadjusted analyses were congestive heart failure (OR: 4.28, 95% CI 2.55–7.18), arrhythmias (OR: 2.86, 95% CI 1.6–5.13), acute myocardial infarction (OR: 2.58, 95% CI 1.52–4.38), moderate or severe renal disease (OR: 2.08, 95% CI 1.07–4.04), peripheral vascular disease (OR: 1.94, 95% CI 1.1–3.4), and hypertension (OR: 1.66, 95% CI 1.12–2.46). The odds ratios of patient comorbidities are described in Table 3. In contrast, other comorbidities such as cerebrovascular disease, dementia, diabetes mellitus, hypothyroidism, obesity, or smoking did not reach statistical significance. The comorbidome of the population can be visualized in Figure 2.

## 4. Discussion

In this study, the comorbidity map of Colombian COPD was described and analyzed, where the relationship between comorbidities and their prevalence was graphically illustrated. The results showed that in the general population, the most prevalent diseases were hypertension and smoking. Additionally, a significant association with mortality was found in comorbidities such as congestive heart failure, arrhythmias, acute myocardial infarction, moderate or severe renal disease, peripheral vascular disease, and hypertension. These findings highlight the importance of considering comorbidities in risk assessment in patients with COPD.

In our cohort, hypertension was the most prevalent comorbidity. This is consistent with the findings of Cordeiro dos Santos et al. [4], who in a systematic review reported a prevalence of 17–64.7%. Regarding its association with mortality, the SUMMIT study [19] demonstrated that elevated systolic blood pressure (HR 1.27, 95% CI 1.12–1.45) and diastolic blood pressure (HR 1.35, 95% CI 1.14–1.59) were associated with increased all-cause mortality. This can be explained by the fact that chronic systemic inflammation and oxidative stress associated with COPD have been associated with decreased nitric oxide bioavailability and endothelial dysfunction, which may contribute to arterial stiffness and hypertension [20].

On the other hand, smoking was also prevalent as reported in previous studies [21,22,23]. These findings suggest that smoking cessation may be an important therapeutic target in COPD management. The relatively lower prevalence of smoking in our cohort may reflect the contribution of other risk factors, such as biomass exposure and environmental pollutants, which are relevant in certain populations.

Congestive heart failure was the comorbidity with the strongest association with mortality in patients with COPD. Divo et al. [12] demonstrated in a multicenter cohort that HF increases the risk of death (HR 1.33, 95% CI: 1.06–1.68) after adjusting for age, sex, and COPD severity. This increased risk can be explained by shared pathophysiological mechanisms between both conditions. COPD has been associated with pulmonary hypertension and right ventricular remodeling, in addition to inducing chronic systemic neurohormonal activation and oxidative stress, contributing to the progressive deterioration of myocardial function and cardiopulmonary dysfunction [24,25,26]. Furthermore, a history of smoking has been described as a common risk factor contributing to coronary dysfunction, thereby increasing the likelihood of developing heart failure [27].

Similarly, arrhythmia is an independent risk factor for mortality in COPD. Divo et al. [12], in a multicenter cohort, found that the presence of arrhythmias was associated with an increased risk of death (HR 1.45, 95% CI 1.12–1.87) after adjusting for age, sex, BMI, and COPD severity. This increased risk may be explained by pathophysiological mechanisms shared with cardiovascular disease, where chronic hypoxemia and systemic inflammation have been associated with atrial remodeling, autonomic dysfunction, and oxidative stress, increasing electrical vulnerability and favoring potentially fatal arrhythmic episodes [28,29,30,31]. In addition, right ventricular overload due to pulmonary hypertension associated with COPD has been associated with atrial dilation, increasing the formation of ectopic foci and reentry circuits [32].

On the other hand, acute myocardial infarction showed a strong association with COPD, which is consistent with the findings of Rothnie et al. [33], who found that a history of acute myocardial infarction increases the risk of mortality in patients with COPD (HR 1.26, 95% CI: 1.13–1.40). This increased risk is attributed to the fact that COPD has been associated with persistent systemic inflammation, sustained oxidative stress, and chronic endothelial dysfunction—mechanisms that may contribute to coronary plaque instability and may impair remodeling and ventricular recovery after the ischemic event, worsening cardiopulmonary function [3,34].

Renal disease was also a risk factor for mortality in patients with COPD. Liu et al. [35], in a systematic review with meta-analysis that included 23 studies, reported that renal disease increases the likelihood of long-term death in this population (OR 1.70, 95% CI: 1.35–2.15). In COPD, chronic systemic inflammation, oxidative stress, intermittent hypoxia, and sustained activation of the renin–angiotensin–aldosterone system have been associated with progressive renal damage, including glomerular fibrosis and microcirculatory impairment. In turn, renal dysfunction has been associated with fluid retention, acid-base imbalances, and accumulation of uremic toxins, which compromise pulmonary and cardiovascular function. This bidirectional interaction may contribute to multisystem deterioration and is associated with a higher risk of mortality [36].

Likewise, peripheral vascular disease has also been described as a relevant comorbidity in patients with COPD. In the population-based study by Terzikhan et al. [37], it was shown that patients with COPD are at risk of developing PVD (OR 1.9, 95% CI 1.1–3.2). However, no statistically significant interaction was observed between the presence of peripheral vascular disease and COPD regarding the risk of mortality. Nevertheless, the mortality rate was higher in patients with both conditions (30.1 per 100,000 person-years) compared to those with only one of them, suggesting a clinically relevant effect. In our cohort, comorbidities such as cerebrovascular disease, dementia, diabetes mellitus, hypothyroidism, obesity, or smoking did not reach statistical significance in their association with mortality. This study provides a comorbidome-based representation of COPD in a Latin American population, integrating prevalence and association with mortality in a visual framework.

Our findings are consistent with global reports. According to the latest GOLD report, COPD is frequently associated with multiple comorbidities that significantly influence prognosis and mortality. Cardiovascular disease, diabetes, and chronic kidney disease have been consistently identified as key contributors to adverse outcomes in COPD populations [38]. In this context, the distribution of comorbidities observed in our cohort aligns with these global patterns, although local factors such as environmental exposures and healthcare access may influence their prevalence.

### Limitations

Among the limitations of our study is its observational nature, with information obtained from clinical records, which may present missing data. However, measures were implemented to minimize information bias, such as continuous training of the staff in charge of data collection. As this research was carried out in a single center, it may have limitations regarding the extrapolation of results; nevertheless, we believe the sample size achieved can support the conclusions obtained. Additionally, some low-frequency comorbidities did not allow the estimation of odds ratios due to sparse data in one of the groups

The design of our study prevents establishing causal relationships between COPD and comorbidities. Although we detected significant associations, the directionality of these relationships remains uncertain. These findings are based on unadjusted associations and should not be interpreted as independent effects. Likewise, data collection through medical records may have limited information about prior treatment or control of comorbidities, which may influence the interpretation of the association.

Comorbidity-specific therapies, such as optimized cardiovascular treatment or interventional procedures, may influence survival in patients with COPD. However, detailed information on these interventions was not consistently available in our dataset, which limits further evaluation of their impact. Temporal changes in clinical management and healthcare delivery may influence hospitalization rates and mortality in COPD populations. Future studies could explore differences across time periods to better understand these trends and their implications for risk stratification.

Information on COPD severity, including GOLD classification or spirometric parameters, was not consistently available, which may confound the observed associations with mortality. Although smoking is a key risk factor for COPD, it was not significantly associated with mortality in our cohort. This finding should be interpreted with caution and may reflect residual confounding or cohort-specific characteristics. The observed inverse association between asthma and mortality is notable and may reflect differences in patient characteristics or disease phenotype, such as asthma-COPD overlap, earlier diagnosis, or closer clinical follow-up. This finding should be interpreted with caution and warrants further investigation.

The extended study period (2005–2020) may introduce temporal heterogeneity, as changes in diagnostic criteria and clinical management over time could have influenced the results. Prospective studies are recommended to investigate etiological relationships between COPD and comorbidities, especially cardiovascular diseases, along with a detailed evaluation of treatment adherence.

## 5. Conclusions

The most prevalent comorbidities were arterial hypertension and smoking. However, the comorbidities significantly associated with mortality were congestive heart failure, arrhythmias, acute myocardial infarction, moderate or severe renal disease, peripheral vascular disease, and arterial hypertension. These findings highlight the clinical relevance of actively identifying and managing cardiovascular comorbidities in risk assessment and therapeutic management of patients with COPD.

## Figures and Tables

**Table 1:** General characteristics of the population.

Variables	General Population n = 500	Alive n = 358	Dead n = 142	*p* Value
Age in years, mean (SD)	76.6 (11.3)	74.3 (11.32)	82.6 (8.81)	<0.001
Age > 65 years, n (%)	428 (85.6)	289 (80.7)	139 (97.9)	<0.001
Male sex, n (%)	288 (57.6)	195 (54.5)	93 (65.5)	0.024
**Residence area, n (%)**				
Rural, n (%)	79 (15.8)	61 (17)	18 (12.7)	0.175
**Comorbidities, n (%)**				
Acute myocardial infarction, n (%)	66 (13.2)	35 (9.8)	31 (21.8)	<0.001
Congestive heart failure, n (%)	72 (14.4)	31 (8.7)	41 (28.9)	<0.001
Peripheral vascular disease, n (%)	58 (11.6)	34 (9.5)	24 (16.9)	0.02
Cerebrovascular disease, n (%)	19 (3.8)	10 (2.8)	9 (6.3)	0.062
Dementia, n (%)	9 (1.8)	4 (1.1)	5 (3.5)	0.068
Connective tissue disease, n (%)	12 (2.4)	11 (3.1)	1 (0.7)	0.119
Peptic ulcer, n (%)	11 (2.2)	8 (2.2)	3 (2.1)	0.933
Mild liver disease, n (%)	1 (0.2)	0	1 (0.7)	0.178
Diabetes without complications, n (%)	67 (13.4)	48 (13.4)	19 (13.4)	0.993
Diabetes with organ damage, n (%)	6 (1.2)	3 (0.8)	3 (2.1)	0.238
Moderate or severe kidney disease, n (%)	39 (7.8)	22 (6.1)	17 (12)	0.028
Arrhythmias, n (%)	52 (10.4)	26 (7.3)	26 (18.3)	<0.001
Hypothyroidism, n (%)	92 (18.4)	60 (16.8)	32 (22.5)	0.133
Hypertension, n (%)	226 (45.2)	149 (41.6)	77 (54.2)	0.011
Obesity, n (%)	31 (6.2)	20 (5.6)	11 (7.7)	0.366
Deficiency anemia, n (%)	4 (0.8)	0	4 (2.8)	0.007
Psychosis, n (%)	2 (0.4)	2 (0.6)	0	0.633
Asthma, n (%)	59 (11.8)	53 (14.8)	6 (4.2)	<0.001
Exacerbations per year, mean (SD)	1.3 (1.29)	1.1 (1.04)	1.6 (1.59)	<0.001
Total number of exacerbations, mean (SD)	2.5 (3.51)	2.4 (3.82)	2.8 (2.85)	0.201
≥2 exacerbations, n (%)	70 (14)	35 (9.8)	35 (24.6)	<0.001
**Exposure history**				
Smoking, n (%)	190 (38)	122 (34.1)	68 (47.9)	0.022
Dust, n (%)	44 (8.8)	35 (9.8)	9 (6.3)	0.047
Fumes, n (%)	17 (3.4)	12 (3.4)	5 (3.5)	0.069
Gases, n (%)	25 (5)	19 (5.3)	6 (4.2)	0.056
Chemicals, n (%)	31 (6.2)	23 (6.4)	8 (5.6)	0.076
Wood smoke, n (%)	134 (26.8)	97 (27.1)	37 (26.1)	0.203
SD: Standard deviation				

**Table 2:** Population prevalences.

Variables	General Population n = 500	Alive n = 358	Dead n = 142
Acute Myocardial Infarction	13.20%	9.78%	21.83%
Congestive Heart Failure	14.40%	8.66%	28.87%
Peripheral Vascular Disease	11.60%	9.50%	16.90%
Cerebrovascular Disease	3.80%	2.79%	6.34%
Dementia	1.80%	1.12%	3.52%
Connective Tissue Disease	2.40%	3.07%	0.70%
Peptic Ulcer Disease	2.20%	2.23%	2.11%
Mild Liver Disease	0.20%	0.00%	0.70%
Diabetes without complications	13.40%	13.41%	13.38%
Diabetes with end-organ damage	1.20%	0.84%	2.11%
Moderate or Severe Renal Disease	7.80%	6.15%	11.97%
Arrhythmias	10.40%	7.26%	18.31%
Hypothyroidism	18.40%	16.76%	22.54%
Hypertension	45.20%	41.62%	54.23%
Obesity	6.20%	5.59%	7.75%
Deficiency Anemia	0.80%	0.00%	2.82%
Psychosis	0.40%	0.56%	0.00%
Asthma	11.80%	14.80%	4.23%
Smoking	38.00%	34.08%	47.89%

**Table 3:** Odds Ratios of the Population.

Variables	Odds Ratio	95% CI
Acute Myocardial Infarction	2.58	1.52–4.38
Congestive Heart Failure	4.28	2.55–7.18
Peripheral Vascular Disease	1.94	1.10–3.40
Cerebrovascular Disease	2.35	0.94–5.92
Dementia	3.23	0.85–12.21
Connective Tissue Disease	0.22	0.03–1.75
Peptic Ulcer Disease	0.94	0.25–3.61
Mild Liver Disease	*	*
Diabetes without complications	1	0.56–1.77
Diabetes with end-organ damage	2.55	0.51–12.81
Moderate or Severe Renal Disease	2.08	1.07–4.04
Arrhythmias	2.86	1.60–5.13
Hypothyroidism	1.44	0.89–2.34
Hypertension	1.66	1.12–2.46
Obesity	1.42	0.66–3.04
Deficiency Anemia	*	*
Psychosis	*	*
Asthma	0.25	0.11–0.60
Smoking	1.5	0.94–2.38

*: Odds ratios could not be estimated due to zero or very low frequency of events in one of the comparison groups. CI: Confidence Interval.

## Data Availability

The data that support the findings of this study are available from the corresponding author upon reasonable request.

## Abbreviations

The following abbreviations are used in this manuscript:

COPD	Chronic Obstructive Pulmonary Disease
CVD	Cardiovascular Disease
CHF	Congestive Heart Failure
AMI	Acute Myocardial Infarction
PVD	Peripheral Vascular Disease
CVA	Cerebrovascular Accident
DEM	Dementia
DMNC	Diabetes Mellitus without Complications
DMC	Diabetes Mellitus with Organ Damage
CKD	Chronic Kidney Disease
ARR	Arrhythmias
HYPOT	Hypothyroidism
HTN	Hypertension
OBE	Obesity
PSY	Psychosis
SMK	Smoking
SD	Standard Deviation
CI	Confidence Interval
OR	Odds Ratio
GOLD	Global Initiative for Chronic Obstructive Lung Disease
FEV1	Forced Expiratory Volume in One Second
FVC	Forced Vital Capacity
REDCap	Research Electronic Data Capture
ADRES	Administrator of Health System Resources
MI	Myocardial Infarction
